# Postpandemic fluctuations of regional respiratory syncytial virus hospitalization epidemiology: potential impact on an immunization program in Switzerland

**DOI:** 10.1007/s00431-024-05785-z

**Published:** 2024-09-27

**Authors:** Klara Fischli, Nina Schöbi, Andrea Duppenthaler, Carmen Casaulta, Thomas Riedel, Matthias V. Kopp, Philipp K. A. Agyeman, Christoph Aebi

**Affiliations:** 1 Division of Pediatric Infectious Disease, Department of Pediatrics, Bern University Hospital, Inselspital, University of Bern, CH-3010 Bern, Switzerland; 2 Division of Pediatric Respiratory Medicine, Department of Pediatrics, Bern University Hospital, Inselspital, University of Bern, Bern, Switzerland; 3 Division of Pediatric Intensive Care Medicine, Department of Pediatrics, Bern University Hospital, Inselspital, University of Bern, Bern, Switzerland; 4 Airway Research Center North (ARCN), Member of the German Lung Research Center (DZL), University of Lübeck, Lübeck, Germany

**Keywords:** Respiratory syncytial virus, Epidemiology, Hospitalization, Vaccine, Nirsevimab, COVID-19

## Abstract

RSV hospitalization epidemiology is subject to rapid changes brought about by the COVID-19 pandemic and the prospect of vaccine prevention. The purpose of this report is to characterize recent epidemiologic and clinical fluctuations and to analyze their potential impact on an immunization program with nirsevimab. This is a 2018–2024 retrospective analysis of all hospitalizations caused by RSV in patients below 16 years of age occurring at an academic Children’s Hospital that serves a defined population. We simulated the vaccine impact against RSV hospitalization by applying the expected effects of the infant immunization program with nirsevimab proposed in Switzerland to observed case counts. We analyzed 1339 hospitalizations. The consecutive occurrence of two major epidemics in 2022–2023 and 2023–2024 had never been recorded previously. The 2023–2024 season witnessed a major shift to older age. Only 61% of patients were below 12 months of age, while prepandemic long-term surveillance since 1997 found a range between 64 and 85% (median, 73%). Age below 3 months, prematurity, airway anomalies, congenital heart disease, and neuromuscular disorders were independently associated with ICU admission. Simulation of the vaccine impact using two scenarios of coverage and efficacy (scenario 1, 50% and 62%, respectively; scenario 2, 90% and 90%) and three different age distributions resulted in an infant vaccine impact of 31.0% (scenario 1) and 81.0% (scenario 2), respectively. Vaccine impact for all patients below 16 years ranged from 22.7 to 24.9% (scenario 1) and 54.2 to 68.8% (scenario 2).

*Conclusion*: RSV hospitalization epidemiology was characterized by substantial variability in patient age on admission. As the proposed RSV immunization program primarily targets infants, year-to-year fluctuation of cases among older children will cause a variability of vaccine impact of approximately 15%. This information may be useful for physicians and hospital administrators when they anticipate the resources needed during the winter season.

**Table:** 

**What is Known:** • *RSV hospitalization epidemiology was subject to massive disturbances during the COVID-19 pandemic.* • *Extended half-life monoclonal antibodies and active maternal immunization offer new means of passive protection of infants against severe RSV disease.* **What is New:** • *We demonstrate substantial year-to-year fluctuation of the age distribution at the time of RSV hospitalization.* • *Up to 40% of annual RSV hospitalizations in a given season occur in children above 12 months of age who do not benefit from maternal RSV immunization and may not be eligible for receipt of a monoclonal antibody.*

**Supplementary Information:**

The online version contains supplementary material available at 10.1007/s00431-024-05785-z.

## Introduction

Respiratory syncytial virus (RSV) is the most common agent causing acute lower respiratory tract infections in children below 5 years of age. It is estimated to cause 60–90% of all cases of bronchiolitis, over 3 million hospitalizations, and over 100,000 deaths worldwide per year [1]. Most children experience their first RSV infection in the first 2–3 years of life [2]. Approximately 1–3% of the annual birth cohort require hospital admission when infected for the first time [3, 4]. Pre-existing chronic conditions such as prematurity, chronic lung disease, congenital heart disease, immunodeficiency, neuromuscular disorders, or age below 3 months increase the risk for severe and complicated courses [5].

Both the epidemiology and the disease burden of RSV became ascertainable and monitorable when testing in nasopharyngeal secretions became routinely available in the mid-1980s [6–8]. At our site, long-term surveillance identified a stable biannual pattern of early-onset high-frequency and late-onset low-frequency season, which also occurs in other locations [4, 9–11]. Within a few years, however, two recent developments led and will lead to drastic changes. First, the COVID-19 pandemic and the associated protective measures at a population level (non-pharmaceutical interventions, NPI) resulted in a massive disruption of the faithfully predictable winter epidemics in temperate climates. In some locations, the first intrapandemic season in 2020–2021 was strongly suppressed, while it was displaced in other locations [12]. The 2021–2022 season again was displaced from winter to spring and summer at some sites while unusual in other ways (e.g., unusual age distribution) in other places. The 2022–2023 season remained unpredictable in many parts of Europe [13–17]. Second, two vaccines for passive [18] and active maternal immunization [19] for the prevention of RSV disease in infants have been approved and entered clinical use in some selected countries in 2023 [20–22]. In Switzerland, a 2024 multidisciplinary consensus statement endorsed by the Federal Office of Public Health proposes an immunization program with the monoclonal antibody nirsevimab that targets all infants intended to prevent RSV infection in the first year of life [23]. As an alternative, a recommendation for active maternal immunization at 32–36 weeks of gestation is expected in the near future [22].

These recent developments require a new perspective on both the epidemiology and the clinical burden of RSV hospitalizations. The aims of the present study were thus twofold. First, we aim to describe the most recent clinical characteristics of RSV hospitalizations analyzing a 6-year period beginning 2 years before the onset of the COVID-19 pandemic and ending 2 years after the last NPI had been lifted, including the 2023–2024 season. Second, we describe two different scenarios of the putative vaccine impact of the proposed nirsevimab immunization program [23], examining how the year-to-year fluctuation of RSV epidemiology as observed since 1997 may affect the in-patient burden of disease remaining.

## Methods

### Setting and case catchment

This is a single-center retrospective clinical analysis of all cases of patients below 16 years of age admitted to our in-patient service between 1 July 2018 and 29 February 2024 with community-acquired RSV infection (core sample). For long-term analysis of the variability of the age at RSV hospitalization, we also used data from our RSV routine surveillance program since 1 July 1997 (long-term sample, see below).

Our institution is the sole provider of primary to tertiary pediatric in-patient services for a general population of approximately 1.2 million and an average annual birth cohort from 2018 to 2024 of 9619 (table S0; Federal Office of Statistics, www.bfs.admin.ch). Case identification consisted of (1) the routine ongoing surveillance program for RSV hospital admissions, (2) electronic text searching of the medical records for “RSV” in the list of main diagnoses of all discharge letters, and (3) searching the clinical microbiology database for all positive RSV test results obtained from nasopharyngeal samples.

Cases were excluded if general informed consent for the use of routine chart data was declined by the parents, if a positive RSV test was first obtained greater than 48 h after admission, or if rehospitalization for RSV infection occurred less than 30 days after a previous RSV admission.

### RSV routine surveillance program

Since 1 July 1997 (except the 2008–2009 RSV season) we have conducted an ongoing, purely observational long-term surveillance of all RSV admissions at our institution [9, 11, 13, 24], in which age, sex, admission date, and date of RSV testing are recorded. The latter has been part of the diagnostic algorithm for patients below 5 years of age admitted with acute-onset respiratory disease. The data on patient age (long-term sample) were used for comparison with the 2018–2024 core sample.

### Definitions

A RSV hospitalization was defined as an admission to the in-patient service of greater than 24 h duration, for which the discharge summary identified an acute upper respiratory tract infection (URTI), wheezy bronchitis, bronchiolitis, or pneumonia as the main diagnosis and a nasopharyngeal sample tested positive for RSV by direct immunofluorescence [25], polymerase chain reaction (PCR), or rapid antigen test [26]. An epidemiological year was defined as beginning on 1 July. The term “vaccine impact” was defined as the percent reduction of RSV hospitalizations associated with a hypothetical RSV immunization program compared with observed case counts. The term “incidence” was defined as RSV hospitalizations per epidemiological year per 100 live births occurring in the catchment area of our institution in the epidemiological year when the patients were born. Table S0 displays the raw birth rate data used. Death due to RSV was defined as a fatal outcome of any cause associated with a RSV hospitalization.

### Data retrieval and analysis

For the 2018 to 2024 core sample, the following variables were retrieved from the electronic medical record of each patient: age, sex, admission and discharge dates, date and time of positive RSV test(s), gestational age, birth weight, pre-existing conditions, main respiratory diagnosis provided by the treating physicians, type of administration of supplemental O_2_, maximum C-reactive protein (CRP) value (mg/L), administration of antibiotics, indication for antibiotic therapy noted by the physicians in charge, intensive care unit (ICU) admission, and death. These data were analyzed for all cases combined and for each RSV season separately. For the 1997–2018 long-term sample, the age and date of RSV admission were retrieved.

### Simulation of RSV vaccine impact

In an attempt to describe the effect of age distributions that varied from season to season on the estimated impact of an immunization program with a long-acting monoclonal antibody (i.e., nirsevimab), we applied two different scenarios to the real-life hospitalization counts. The hypothetical vaccination program made nirsevimab available to all infants for protection during their first RSV season in life and to children with at least one of the pre-existing conditions listed in Table 1 for their second RSV season. The real-life data consisted of the 2017–2018 season (i.e., the last season of the long-term sample) (shift to younger age), the 2019–2020 season (average age distribution), and the 2023–2024 season (shift to older age). The estimated vaccine impact (%) was calculated as vaccine coverage × vaccine efficacy × 100. Hypothetical scenario 1 used an infant vaccine coverage of 50%, simulating year 1 of an immunization program, and the vaccine efficacy of nirsevimab of 62% [18]. The respective figures for scenario 2 are 90% and 90%. The greater efficacy figure used reflects recent data from the HARMONIE trial, a European multicenter study [27], and early real-world effectiveness data [28–30]. Vaccine efficacy was considered identical for all hypothetically eligible patients. Patients with pre-existing illnesses were considered 100% vaccinated.

**Table 1:** Clinical characteristics of 1339 patients hospitalized because of a RSV infection from 2018 to 2024 at the Department of Pediatrics, Bern University Hospital

	2018–2024		2018–2019		2019–2020		2021–2022		2022–2023		2023–2024	
	*n*	%	*n*	%	*n*	%	*n*	%	*n*	%	*n*	%
Demography												
Cases extracted (*n*)	1410		237		182		345		301		345	
Cases excluded (*n*)	71	5.0	1		2		39		2		27	
Cases included (*n*)	1339		236		180		306		299		318	
Male sex (*n*)	737	55.0	119	50.4	110	61.1	174	56.9	158	52.8	176	55.3
Median age (years) [IQR]	0.46 [0.15–1.25]		0.44 [0.15–1.21]		0.39 [0.13–1.02]		0.52 [0.16–1.39]		0.30 [0.12–1.09]		0.68 [0.21–1.70]	
Age < 3 months	495	37.0	94	39.8	72	40.0	103	33.7	133	44.5	93	29.2
Age < 6 months	698	52.1	136	57.6	101	56.1	148	48.4	176	58.9	138	43.4
Age < 12 months	915	68.3	172	72.9	134	74.4	205	67.0	211	70.6	193	60.7
Age 12–23 months	236	17.6	43	18.2	30	16.7	51	16.7	52	17.4	60	18.9
Age 24–59 months	155	11.6	16	6.8	16	8.9	46	15.0	29	9.7	48	15.1
Age ≥ 60 months	33	2.5	5	2.1	0		4	1.3	7	2.3	17	5.3
Gestational age (*n* = 1165)												
< 32 weeks	53	4.5	15	6.9	5	3.1	12	4.4	9	3.3	12	5.0
32–36 weeks	130	11.2	37	17.1	18	11.0	25	9.1	22	8.1	28	11.6
≥ 37 weeks	982	84.3	164	75.9	140	85.9	237	86.5	239	88.5	202	83.5
Birth weight (*n* = 1098)												
< 750 g	16	1.5	6	2.9	1	0.6	2	0.8	4	1.6	3	1.4
750–1499 g	38	3.5	11	5.3	3	1.9	9	3.5	6	2.4	9	4.1
1500–2499 g	102	9.3	26	12.6	16	10.0	18	6.9	20	7.9	22	10.1
≥ 2500 g	942	85.8	164	79.2	140	87.5	231	88.8	223	88.1	184	84.4
Pre-existing conditions												
None	1084	81.0	188	79.7	143	79.4	246	80.4	245	81.9	262	82.4
Bronchopulmonary dysplasia	31	2.3	10	4.2	5	2.8	6	2.0	5	1.7	5	1.6
Laryngeal or tracheobronchial anomaly	24	1.8	3	1.3	4	2.2	3	1.0	5	1.7	9	2.8
Any respiratory tract condition	159	11.9	33	14.0	26	14.4	41	13.4	29	9.7	30	9.4
Congenital heart disease^1^	24	1.8	4	1.7	3	1.7	5	1.6	7	2.3	5	1.6
Neuromuscular disease	26	1.9	5	2.1	0		2	0.7	11	3.7	8	2.5
Immunocompromised state or malignancy	13	1.0	2	0.8	2	1.1	3	1.0	4	1.3	2	0.6
Down syndrome	14	1.0	1	0.4	3	1.7	3	1.0	6	2.0	1	0.3
Other	60	4.5	9	3.8	5	2.8	1	0.3	16	5.4	19	6.0
≥ 1 condition	255	19.0	48	20.3	37	20.6	60	19.6	54	18.1	56	17.6
In-hospital diagnosis and management												
Main discharge diagnosis												
Upper respiratory tract infection	96	7.2	17	7.2	13	7.2	29	9.5	13	4.3	24	7.5
Wheezy bronchitis	127	9.5	14	5.9	16	8.9	42	13.7	27	9.0	28	8.8
Bronchiolitis	949	70.9	181	76.7	133	73.9	212	69.3	233	77.9	190	59.7
Pneumonia	167	12.5	24	10.2	18	10.0	23	7.5	26	8.7	76	23.9
Supplemental oxygen administration												
None	227	17.0	47	19.9	36	20.0	66	21.6	33	11.0	45	14.2
Nasal cannula or funnel	755	56.4	137	58.1	109	60.6	164	53.6	174	58.2	171	53.8
High flow nasal cannula	301	22.5	43	18.2	33	18.3	60	19.6	82	27.4	83	26.1
CPAP	33	2.5	1	0.4	1	0.6	11	3.6	7	2.3	13	4.1
Mechanical ventilation	23	1.7	8	3.4	1	0.6	5	1.6	3	1.0	6	2.0
Antimicrobial therapy for all indications	317	23.7	59	25.0	45	25.0	77	25.2	61	20.4	75	23.6
Acute otitis media	133	9.9	20	8.5	20	11.1	34	11.1	28	9.4	31	9.7
Pneumonia	106	7.9	23	9.7	13	7.2	23	7.5	17	5.7	30	9.4
Sepsis or fever without source	47	3.5	12	5.1	7	3.9	13	4.2	8	2.7	7	2.2
Other or unrelated to RSV	31	2.3	4	1.7	5	2.8	7	2.3	8	2.7	7	2.2
None	1022	76.3	177	75.0	135	75.0	229	74.8	238	79.6	243	76.4
Outcome												
Hospital stay (d)	4.6 [3.0–7.0]		4.5 [3.0–6.4]		4.0 [3.0–6.0]		4.2 [3.0–6.4]		5.0 [3.0–7.0]		5.0 [3.0–7.0]	
ICU admission (*n*)	152	11.4	29	12.3	16	8.9	30	9.8	37	12.4	40	12.6
Death	2	0.1	0		0		0		0		2	0.6

^1^Hemodynamically significant at the time of RSV hospitalization

### Statistical analysis and software used

Data are presented as frequencies and proportions for categorical variables and as medians and interquartile ranges (IQRs) or ranges for continuous variables. Categorical variables were compared with the *χ*^2^ test or Fisher’s exact test. Continuous variables were compared with the Mann–Whitney *U*-test. *p*-values below 0.05 were considered statistically significant. To explore associations between ICU admission and epidemiological or clinical variables, we fitted multivariate binomial regression models. We separately fitted univariable models for the patient sex, gestational age at birth, age at admission with RSV infection, pre-existing chronic diseases (bronchopulmonary dysplasia, laryngeal or tracheobronchial anomaly, congenital heart disease, neuromuscular disease, Down syndrome, or other pre-existing chronic disease), main respiratory tract discharge diagnosis, antibiotic therapy during hospital stay, and respiratory co-infection with another virus. We included variables with a *p*-value < 0.1 from likelihood ratio tests in the final model. We presented the results of the multivariate binomial regression analysis as odds ratios (OR) with 95% confidence intervals and *p*-values from likelihood ratio tests. Statistical analyses were performed using the R software package, version 4.0.3 (Vienna, Austria; https://www.R-project.org/).

## Results

### Time sequence of RSV hospitalizations 2018–2024 (core sample)

**Fig. 1:**
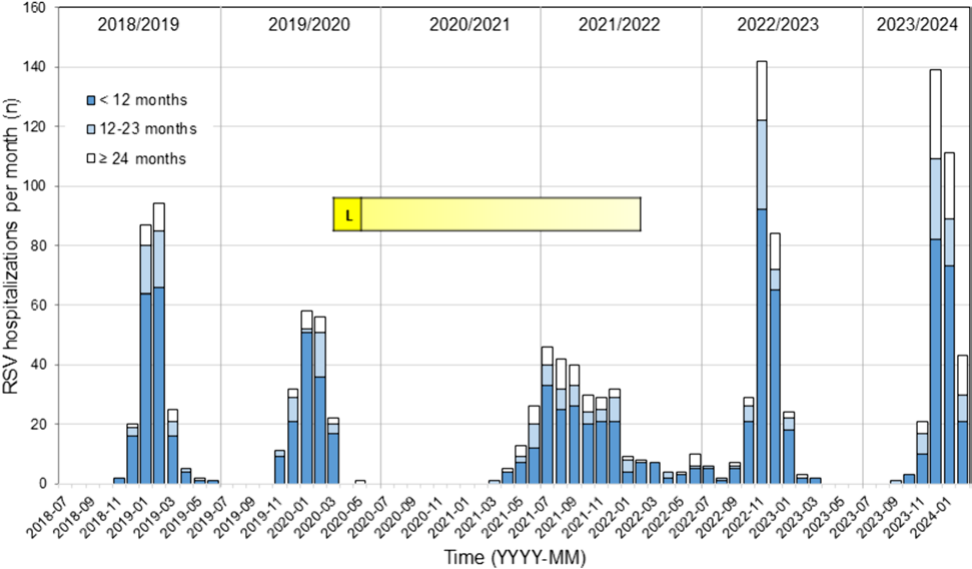
Monthly RSV hospitalization frequencies between 1 July 2018 and 29 February 2024 at the Department of Pediatrics, Bern University Hospital, for children below 12 months, 12–23 months, and ≥ 24 months of age, respectively. The yellow bar marks the time period of the lockdown (“L”) related to the COVID-19 pandemic and the total time span of population-wide non-pharmaceutical interventions (NPI)

Figure 1 depicts the monthly case counts comprising a total of 1339 RSV hospitalizations that occurred during the 6-year study period. The yellow bar indicates the timespan of COVID-19 NPI. Complete lockdown (L) lasted for 2 months beginning on 16 March 2020. The implementation of NPIs coincided with an abrupt cessation of cases and complete suppression of RSV hospitalizations in the winter of 2020–2021. The subsequent out-of-season epidemic in 2021–2022 started in March 2021, reached a peak in July 2021, and ended in the summer of 2022. Monthly case counts throughout this season were comparatively low but activity never entirely ceased until the 2022–2023 season took off in November 2022. It was followed by an equally strong 2023–2024 season that peaked 13 months after the preceding season’s peak.

### Age distribution

**Fig. 2:**
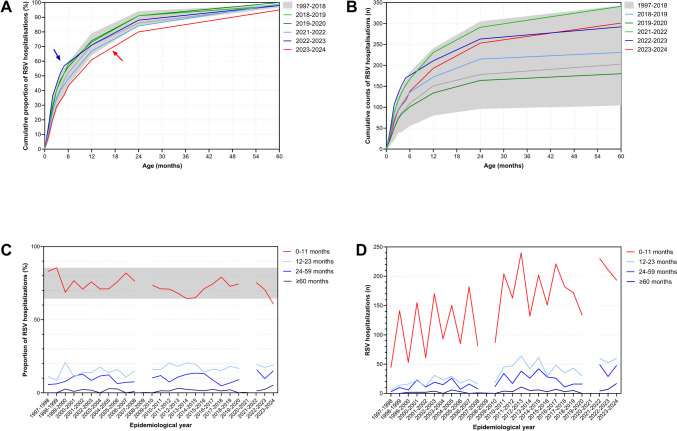
**A**, **B** Cumulative proportions (%) and cumulative case counts (*n*) of RSV hospitalizations per epidemiological year between 2018 and 2024 on the *y*-axis as a function of the age at admission. The gray lines and areas show the median and range of all RSV seasons from 1997 to 2018. The 2022–2023 season is notable for a large proportion of cases below 3 months of age (blue arrow). The age distribution of the 2023–2024 season is entirely shifted to the right (red arrow). Time series of the proportions (**C**) and absolute case counts (**D**) of annual cases in the respective age groups from 1997 to 2024. The gray area depicts the range of proportions below 12 months of age from 1997 to 2018. The left vertical gap identifies a year of missing surveillance data (2008–2009); the right gap identifies the first pandemic year, when no cases were observed (2020–2021)

Figure 2 illustrates the season-to-season variability of the age distribution at RSV hospitalization during the five core seasons from 2018 to 2024 in comparison with the range of observations between 1997 and 2018 (Fig. 2A and 2B). Most notable was a relative increase of cases in patients below 3 months of age in the 2022–2023 season and, conversely, a major shift of the entire curve to older age in the 2023–2024 season. While a median proportion of 73% (range, 64–86%) of patients were 0–11 months of age at admission in 1997–2018, the respective figure in the 2023–2024 season was 61%. Thus, 39% of children admitted because of a RSV infection in that season were older than 12 months of age (Fig. 2C and 2D).

**Fig. 3:**
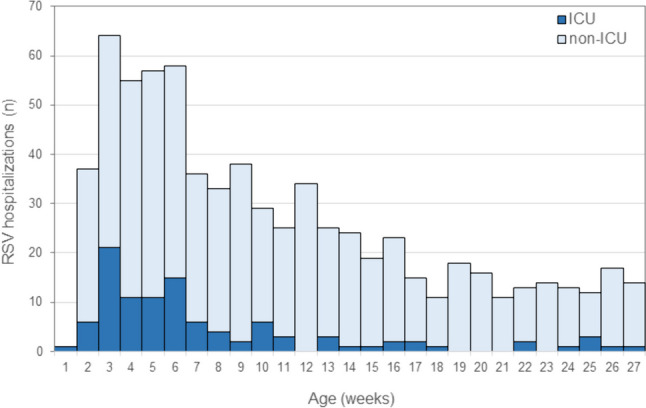
Histogram of weekly RSV hospitalization frequencies according to chronological age in weeks of 713 infants below 27 weeks of age admitted to the Department of Pediatrics, Bern University Hospital between 2018 and 2024. ICU admission counts are shown in dark blue

In addition, the pooled age distribution of the entire 6-year core sample revealed that the nearly complete lack of cases during the first week of life was followed by a rapid increase in weekly case counts reaching a maximum for both general ward and ICU admissions in week three of life (Fig. 3).

### Clinical characteristics of the 2018–2024 core sample

Table 1 summarizes the main clinical characteristics of the entire 2018–2024 core sample and of each of the five individual RSV seasons separately. A total of 71 cases (5%) were excluded because of parental refusal to participate. The data on gestational age and birth weight were nearly complete for patients below 1 year of age but became increasingly incomplete with advancing age at admission (see Table 2).

**Table 2:** Average annual incidences in 2018–2024 of RSV hospitalizations per 100 live births according to age, gestational age, and distribution of pre-existing conditions

	0–11 months				12–23 months				24–59 months			
	All hospitalizations		ICU admissions		All hospitalizations		ICU admissions		All hospitalizations		ICU admissions	
	*n*	Incidence (%)^2^	*n*	Incidence (%)^2^	*n*	Incidence (%)^2^	*n*	Incidence (%)^2^	*n*	Incidence (%)^2^	*n*	Incidence (%)^2^
All cases	915	1.59	114	0.20	236	0.40	21	0.04	155	0.09	12	0.01
Cases by gestational age^1^												
< 32 weeks	18	3.12	6	1.04	32	5.41	5	0.85	16	0.88	2	0.11
32–36 weeks	97	3.06	22	0.69	28	0.86	4	0.12	22	0.22	6	0.06
≥ 37 weeks	800	1.48	86	0.16	176	0.32	12	0.02	117	0.07	4	0.002
Pre-existing conditions	*n*	Proportion (%)^3^	*n*	Proportion (%)^3^	*n*	Proportion (%)^3^	*n*	Proportion (%)^3^	*n*	Proportion (%)^3^	*n*	Proportion (%)^3^
None	826	90.3	93	81.6	158	66.7	8	38.1	90	58.1	3	25.0
Bronchopulmonary dysplasia	5	0.5	2	1.8	16	6.8	3	14.3	8	5.2	1	8.3
Laryngeal or tracheobronchial anomaly	10	1.1	2	1.8	8	3.4	3	14.3	3	1.9	1	8.3
Any respiratory tract condition	45	4.9	12	10.5	53	22.4	12	57.1	45	29.0	7	58.3
Congenital heart disease	14	1.5	6	5.3	6	2.5	1	4.8	3	1.9	1	8.3
Neuromuscular disease	3	0.3	2	1.8	9	3.8	1	4.8	8	5.2	1	8.3
Immunocompromised state or malignancy	2	0.2	0	0.0	2	0.8	1	4.8	4	2.6	0	0.0
Down syndrome	5	0.5	2	1.8	3	1.3	0	0.0	5	3.2	2	16.7
Other	21	2.3	6	5.3	17	7.2	2	9.5	14	9.0	2	16.7
≥ 1 condition	89	9.7	21	18.4	78	32.9	13	61.9	65	41.9	9	75.0

Overall, except for the age distribution, the majority of the clinical criteria analyzed were stable and showed little year-to-year fluctuation. Eighty-one percent of cases occurred in patients without pre-existing conditions (Table 1). Among the remaining, half suffered from a respiratory tract condition. Multiple pre-existing conditions were found in 6.6%. None of these conditions fluctuated substantially from season to season. This was also true for the need for and type of supplemental O_2_ administration. Seven percent of admitted children required no supplemental O_2_. These were predominantly very young infants admitted for RSV-related feeding difficulties. However, season-to-season comparison revealed that in 2023–2024 a significantly larger proportion of cases received the diagnosis of pneumonia rather than any of the other three discharge diagnoses. This was associated with older age, but not with clinical evidence suggesting more severe disease in 2023–2024 (table S4). Overall, a stable proportion of approximately 25% of RSV patients received antibiotic therapy, the most common indications being acute otitis media (9.9%) and pneumonia (7.9%).

### Intensive care unit (ICU) admissions

One-hundred and fifty-two patients (11.4%) were admitted to the ICU (clinical dataset available in table S1). Of these, 28 (18.4%) and 23 (15.1%) required continuous positive airway pressure (CPAP) support or mechanical ventilation, respectively. The 2023–2024 season again was unusual in that patients were older (e.g., 40.0% were older than 12 months of age vs. 19.6% in 2018–2023; OR 2.73; 95% CI 1.24–5.98; *p* = 0.012) and that significantly more often the diagnosis of pneumonia was made (30.0% in 2023–2024 vs. 8.9% in 2018–2023; OR 4.37; 95% CI 1.71–11.16; *p* = 0.003). Greater than two-thirds (68.4%) of patients admitted to the ICU were previously healthy. Multiple logistic regression analysis (table S3) identified that chronologic age below 3 months, prematurity below 32 weeks, prematurity of 32 to 36 weeks, laryngeal or tracheobronchial anomalies, hemodynamically significant congenital heart diseases, and neuromuscular disorders were independently associated with ICU admission. ICU patients were also significantly more likely to receive antimicrobial therapy during the course of their hospital stay than patients cared for in general wards only.

### RSV hospitalizations in patients 12–23 months of age

A total of 236 patients (17.7%) were hospitalized in their second year of life. These patients more often suffered from known pre-existing conditions than infants (31.6% vs. 9.7%; OR 4.30; 95% CI 3.03–6.10; *p* < 0.0001). They also received antibiotics more often than infants (33.3% vs. 17.7%; OR 2.32; 95% CI 1.69–3.20; *p* < 0.0001). The ICU admission rates of these two age groups were similar (9.3% vs. 12.5%; OR 0.75; 95% CI 0.44–1-1.16; *p* = 0.216).

### RSV hospitalization incidence

Table 2 lists the estimated average annual RSV hospitalization incidences in our institution’s catchment area for the first, second, and third to fifth years of life. Only 5 RSV waves occurred during the 6-year period covered (Fig. 1). Figure S1 displays the incidence data for each individual year. In addition, Table 2 lists incidences according to three ranges of gestational age. Some data on gestational age were missing, particularly after the age of 1 year. Thus, the absolute figures given for the three gestational age ranges were extrapolated from the raw data assuming that the gestational age distribution was the same for the entire cohort. In the first year of life, the incidences were the same for infants born before 32 weeks of gestation and those born between 32 and 36 weeks. In the second year of life, however, the incidence increased in the former, while it decreased in the latter. This was not true for ICU admissions. Table 2 also lists the absolute relative occurrence of co-morbidities. The proportion of patients with at least one pre-existing illness was greater in ICU admissions than in regular ward admissions and increased with age for both types of admission. Respiratory tract conditions were by far the most common underlying illnesses.

### Simulated RSV vaccine impact

As shown in Table 3, calculations for immunization scenario 1 resulted in an estimated immunization program impact of 20.7–27.6% and, consequently, 72.4–79.3% of cases remaining with minor differences between the three types of seasons. The estimated impact among cases with co-morbidities was somewhat greater because we assumed full vaccine coverage in these patients. Calculations for scenario 2 predicted a vaccine impact of 54.2–68.8% with 31.2–45.8% of cases remaining. The lowest impact was calculated for the 2023–2024 season and reflects the large proportion of patients older than 12 months of age who would not be eligible for vaccination.

**Table 3:** Potential reduction of RSV hospitalizations achieved by the currently proposed Swiss immunization program [23] using the monoclonal antibody nirsevimab

	Cases observed (*n*)			Scenario 1: coverage 50%; study efficacy 62%						Scenario 2: coverage 90%; effectiveness 90%					
				2017–2018		2019–2020		2023–2024		2017–2018		2019–2020		2023–2024	
				Impact		Impact		Impact		Impact		Impact		Impact	
	2017–2018	2019–2020	2023–2024	*n*^b^	%	*n*^b^	%	*n*^b^	%	*n*^b^	%	*n*^b^	%	*n*^b^	%
Age (months)															
< 12	182	134	193	56	31.0	42	31.0	60	31.0	147	81.0	109	81.0	156	81.0
12–23	35	30	60	7	18.6	6	19.2	10	17.4	9	27.0	8	27.9	15	25.2
≥ 24	11	16	65	0	0.0	0	0.0	0	0.0	0	0.0	0	0.0	0	0.0
All	228	180	318	63	27.6	48	26.7	70	22.1	157	68.8	117	65.0	171	53.9
ICU admissions															
< 12	8	11	24	2	31.0	3	31.0	7	31.0	6	81.0	9	81.0	19	81.0
12–23^a^	1	3	10	0.2	24.8	1	19.2	1	5.2	1	81.0	1	25.2	3	25.2
≥ 24	3	2	6	0	0.0	0	0.0	0	0.0	0	0.0	0	0.0	0	0.0
All	12	16	40	3	22.7	4	24.9	8	19.9	7	60.8	10	60.4	22	54.9
With comorbidity															
< 12	17	18	14	11	62.0	11	62.0	9	62.0	15	90.0	16	90.0	13	90.0
12–23	4	9	17	2	62.0	6	62.0	11	62.0	4	90.0	8	90.0	15	90.0
≥ 24	10	10	25	0	0.0	0	0.0	0	0.0	0	0.0	0	0.0	0	0.0
All	31	37	56	13	42.0	17	45.2	20	**35.7**	19	61.0	24	65.7	28	49.8

^a^No. of cases prevented

^b^Proportion of ICU patients with comorbidity: 2017–2018, 40%; 2019–2020, 31%; 2023–2024, 28%

## Discussion

Pediatric epidemiology and prevention of RSV disease have and will face rapid changes during the 2020s. Here, we provide up-to-date regional hospitalization data from the past five RSV epidemics occurring between 2018 and 2024. These data are likely to be useful for monitoring how epidemic cycling will continue to evolve after the pandemic disruption. They also serve as baseline quantitation of the hospital burden of disease before vaccine prevention becomes effective.

The epidemic curve (Fig. 1) demonstrates that—after one out-of-season epidemic in 2021–2022—winter seasonality quickly re-emerged. This unique inter-seasonal epidemic was a Europe-wide phenomenon [13, 16, 31, 32] and marked the reappearance of RSV after 1 year of near-complete suppression of circulation. It began in late spring and reached a peak in the summer of 2021. The ensuing major winter season of 2022–2023 suggested that biannual cycling, i.e., the alternating occurrence of major and minor seasons, would also be re-established. The 2023–2024 season, however, proved to be equally strong and intervened at a shorter interval than expected for biannual cycling [9]. Indeed, modeling studies dating back to as early as 2020 correctly predicted that several years of irregularities would follow, before stable cycling would resume [33].

A post-lockdown age shift toward older age at hospitalization was reported in many studies worldwide, mainly from high-income countries [34]. Older age was typically observed in the first post-lockdown season [14, 16, 35, 36]. In contrast, we failed to find a major shift of age at that time when compared with more than 20 seasons recorded previously (Fig. 2C) but did so in the third post-lockdown season in 2023–2024. Similar observations of delayed age-shifting were most recently reported from St. Etienne, France, for the 2023–2024 season [37] and from Colorado, USA, for the 2022–2023 season [38]. The reasons are unclear. The “immune debt” hypothesis implies that the NPI-mediated lack of first-year-of-life exposure to RSV resulted in more frequent hospitalizations at older age [31, 36, 39]. It appears unlikely, however, to account for a late age shift 2–3 years after the resumption of viral circulation. Methodological reasons may play a role and include more frequent testing in older children [40, 41], the use of PCR rather than less sensitive techniques, and the use of multiplex PCR techniques detecting RSV even if it is not the etiology of the clinical syndrome investigated. Cantais and co-workers postulated that the age shift that they reported from St. Etienne, France, in 2023–2024 could be attributed to the newly implemented nirsevimab vaccination program [37]. In contrast, the data from our center, located at a distance of merely 360 km from St. Etienne, demonstrate that a major shift to older age occurred in the absence of RSV vaccination and did so at the same time as reported from St. Etienne. A recent follow-up study of the MELODY trial [18] following patients, who had received nirsevimab or a placebo in infancy, also failed to record an increase in frequency or severity of RSV infections in the second RSV season of life [42]. It is thus conceivable that the age shift observed in St. Etienne was at least in part unrelated to the use of nirsevimab.

The age at RSV admission on the opposite side of the age spectrum is equally important. Passive immunization using an extended half-life monoclonal antibody requires timely postnatal administration during the RSV season. This may cause a logistic challenge as our data (Fig. 3) emphasize that the administration should be ensured during the first week of life. Weekly case counts peaked in week 3 of life and cannot be lowered optimally if vaccination is delayed. Thus, the future parents’ information and decision process concerning this vaccine should be completed as early as possible. Maternity units will need to offer the vaccine before the babies are discharged home, and babies born in an outpatient setting should be offered the vaccine promptly, e.g., at the time of the blood sampling for the neonatal screening program, either by a pediatrician or by a midwife.

Other than for these age considerations, we found remarkably little season-to-season fluctuation of the clinical variables investigated (Table 1), an exception being the increase in the clinical diagnosis of pneumonia in 2023–2024. This finding appeared to be related to the age shift with a concomitant decrease in the clinical syndrome of bronchiolitis. However, we found no evidence of more severe clinical disease or more frequent bacterial secondary infection (table S4).

The main goal of a RSV immunization program is the prevention of acute respiratory failure resulting in ICU admission. Not surprisingly, our logistic regression analysis identified age below 3 months, prematurity below 32 weeks, prematurity of 32–36 weeks, and the presence of cardiorespiratory and neuromuscular illnesses as independent factors predicting ICU admission (table S3). These findings confirm established knowledge [43, 44]. They emphasize that in the event of a vaccine shortage, which is a realistic scenario as demands may soar quickly, infants with one or more of these risk factors should be offered preferential vaccine access. Conversely, if vaccine supply is unlimited, second-year prevention using a monoclonal antibody may be considered for these risk groups as proposed by the Swiss consensus proposal [23]. Incidence data (Table 2, top) identified prematurity below 32 weeks of gestation as a major risk factor for RSV hospitalization in the second year of life, presumably together with severe cardiorespiratory co-morbidities, neuromuscular disease, and Down syndrome (Table 2, bottom). We did not calculate incidence data for these entities as we lacked reliable population denominators. Late prematurity, on the other hand, was associated with a minor increase compared with birth at term and was not associated with a substantial number of ICU admissions (table S1).

Overall, the bulk of RSV hospitalizations occur in previously healthy children and the impact of an immunization program will be driven by its effect on healthy infants born at term [45]. As recently implemented [20, 21] or proposed [23] vaccination programs primarily target the infant population, the age distribution at RSV admission discussed above is a relevant factor affecting their real-world effectiveness. Our calculations in Table 3 predict that depending on the type of age distribution in a given season, the vaccine impact of nirsevimab may vary by approximately 15% (scenario 2). This gap is most apparent when vaccine coverage among infants is high, and recent data from Spain suggest that this is a conceivable scenario [29]. More importantly, the data show that the absolute case counts remaining will be substantial, particularly in the early years of an immunization program with limited vaccine coverage. In-patient services will thus continue to see substantial numbers of children with RSV disease.

Several limitations of this study need to be addressed. Apart from the retrospective single-center design, the estimated vaccine impact was extrapolated retrospectively from observed case frequencies rather than using, e.g., a test-negative design [46]. Also, the vaccine impact was considered to be stable rather than accounting for the waning of protection over the period of exposure. Ultimately, we could not control for the different performances of RSV testing methods used and for changes in testing practice over the 25-year overall observation period, even though the diagnostic algorithm for RSV testing remained unchanged. In particular, rigid testing and the use of PCR since the beginning of the COVID-19 pandemic were likely to identify more cases among older children and may have contributed to the observed age shift to older age.

In conclusion, this study provides data showing that the COVID-19-related disruption of RSV circulation continues to result in an unpredictable shape of annual winter epidemics. New vaccines offer an unprecedented opportunity to prevent the majority of RSV hospitalizations. Volatility of the age at admission, however, will lead to appreciable fluctuations of vaccine effectiveness, at least until stable epidemic cycling is restored. Hospital pediatricians and administrators are well advised to consider these uncertainties when anticipating the resources needed for the winter months.

## Supplementary Information

Below is the link to the electronic supplementary material.

Supplementary file1 (DOCX 350 KB)

## Data Availability

Data is provided within the manuscript or supplementary information files.

## Abbreviations

CI: Confidence interval
CRP: C-reactive protein
ICU: Intensive care unit
IQR: Interquartile range
NPI: Non-pharmaceutical interventions
OR: Odds ratio
PCR: Polymerase chain reaction
RSV: Respiratory syncytial virus
URTI: Upper respiratory tract infection
