# Supplementary material for: Postpandemic fluctuations of regional respiratory syncytial virus hospitalization epidemiology: potential impact on an immunization program in Switzerland
Source: Eur J Pediatr. 2024 Sep 27;183(12):5149–61. doi: 10.1007/s00431-024-05785-z (PMC11527947; doi:10.1007/s00431-024-05785-z)

Postpandemic Fluctuations of Regional Respiratory Syncytial Virus Hospitalization Epidemiology – Impact on Potential Effectiveness of a RSV Immunization Program in Switzerland

Klara Fischli, Nina Schöbi, Andrea Duppenthaler, Carmen Casaulta, Thomas Riedel, Matthias V Kopp, Philipp KA Agyeman, Christoph Aebi (christoph.aebi@insel.ch)

**Supplementary data file**

| Table S0 ……………. | Live birth rate per epidemiological year used for calculation of RSV hospitalization incidences |
| --- | --- |
| Table S1 ……………. | Clinical characteristics of 152 Intensive Care Unit RSV admissions |
| Table S2 ……………. | Clinical characteristics of 236 RSV admissions among childrend 12 to 23 months of age |
| Table S3 ……………. | Multiple logistic regression analysis of clinical characteristics associated with ICU admission |
| Table S4 ……………. | Comparison of clinical variables among RSV patients diagnosed with pneumonia in 2018-2023 vs. 2023-2024 |
| Figure S1 …………… | Age group-specific incidences of RSV hospitalizations per epidemiologic year |

**Table S0**. Live birth rate per epidemiological year used for calculation of RSV hospitalization incidences

| Epidemiological year | Live births (n) |
| --- | --- |
| 2014-2015 | 9’902 |
| 2015-2016 | 10’093 |
| 2016-2017 | 10’127 |
| 2017-2018 | 10’143 |
| 2018-2019 | 10’039 |
| 2019-2020 | 9’939 |
| 2020-2021 | 10’103 |
| 2021-2022 | 9’770 |
| 2022-2023 | 9’173 |
| 2023-2024 | 8’690 |

Source: Swiss Federal Office of Statistics ([www.bfs.admin.ch](http://www.bfs.admin.ch))

**Table S1.** Clinical characteristics of 151 RSV Intensive Care Unit (ICU) hospitalizations from 2018 to 2024 at the Department of Pediatrics, Bern University Hospital, Bern, Switzerland

|  | **2018-2024** | |  | **2018-2019** | |  | **2019-2020** | |  | **2021-2022** | |  | **2022-2023** | |  | **2023-2024** | |
| --- | --- | --- | --- | --- | --- | --- | --- | --- | --- | --- | --- | --- | --- | --- | --- | --- | --- |
|  | **n** | **%** |  | **n** | **%** |  | **n** | **%** |  | **n** | **%** |  | **n** | **%** |  | **n** | **%** |
| ***Demography*** |  |  |  |  |  |  |  |  |  |  |  |  |  |  |  |  |  |
| All admissions | 1’339 |  |  | 236 |  |  | 180 |  |  | 306 |  |  | 299 |  |  | 318 |  |
| ICU admission (n) | 152 | 11.4 |  | 29 | 12.3 |  | 16 | 8.9 |  | 30 | 9.8 |  | 37 | 12.4 |  | 40 | 12.6 |
| Male sex (n) | 85 | 55.9 |  | 18 | 62.1 |  | 9 | 56.3 |  | 13 | 43.3 |  | 18 | 48.6 |  | 27 | 67.5 |
|  |  |  |  |  |  |  |  |  |  |  |  |  |  |  |  |  |  |
| Median age (years) | 0.16  [0.07-0.97] |  |  | 0.11  [0.07-0.26] |  |  | 0.10  [0.05-1.27] |  |  | 0.12  [0.07-0.49] |  |  | 0.11  [0.06-0.41] |  |  | 0.73  [0.18-1.68] |  |
| age < 3 months | 88 | 57.9 |  | 21 | 72.4 |  | 11 | 68.8 |  | 19 | 63.3 |  | 26 | 70.3 |  | 11 | 27.5 |
| age < 6 months | 103 | 67.8 |  | 23 | 79.3 |  | 11 | 68.8 |  | 22 | 73.3 |  | 28 | 75.7 |  | 19 | 47.5 |
| age < 12 months | 114 | 75.0 |  | 25 | 86.2 |  | 11 | 68.8 |  | 24 | 80.0 |  | 30 | 81.1 |  | 24 | 60.0 |
| age 12-23 months* | 21 | 13.8 |  | 2 | 6.9 |  | 3 | 18.8 |  | 3 | 10.0 |  | 3 | 8.1 |  | 10 | 25.0 |
| age 24-59 months | 12 | 7.9 |  | 1 | 3.4 |  | 2 | 12.5 |  | 3 | 10.0 |  | 2 | 5.4 |  | 4 | 10.0 |
| age ≥60 months | 5 | 3.3 |  | 1 | 3.4 |  | 0 | 0.0 |  | 0 | 0.0 |  | 2 | 5.4 |  | 2 | 5.0 |
|  |  |  |  |  |  |  |  |  |  |  |  |  |  |  |  |  |  |
| Gestational age (n=131 patients) |  |  |  |  |  |  |  |  |  |  |  |  |  |  |  |  |  |
| < 32 weeks | 10 | 7.6 |  | 2 | 7.4 |  | 2 | 12.5 |  | 3 | 11.1 |  | 1 | 3.1 |  | 2 | 6.9 |
| 32-36 weeks | 26 | 19.8 |  | 10 | 37.0 |  | 1 | 6.3 |  | 4 | 14.8 |  | 3 | 9.4 |  | 8 | 27.6 |
| ≥ 37 weeks | 95 | 72.5 |  | 15 | 55.6 |  | 13 | 81.3 |  | 20 | 74.1 |  | 28 | 87.5 |  | 19 | 65.5 |
|  |  |  |  |  |  |  |  |  |  |  |  |  |  |  |  |  |  |
| Birth weight (n=123) |  |  |  |  |  |  |  |  |  |  |  |  |  |  |  |  |  |
| < 750 g | 1 | 0.8 |  | 0 | 0.0 |  | 0 | 0.0 |  | 0 | 0.0 |  | 1 | 3.1 |  | 0 | 0.0 |
| 750-1499 g | 9 | 7.3 |  | 2 | 8.0 |  | 2 | 12.5 |  | 2 | 8.3 |  | 1 | 3.1 |  | 2 | 6.9 |
| 1500-2499 g | 15 | 12.2 |  | 5 | 20.0 |  | 1 | 6.3 |  | 3 | 12.5 |  | 1 | 3.1 |  | 5 | 17.2 |
| ≥ 2500 g | 98 | 79.7 |  | 18 | 72.0 |  | 13 | 81.3 |  | 19 | 79.2 |  | 29 | 90.6 |  | 19 | 65.5 |
|  |  |  |  |  |  |  |  |  |  |  |  |  |  |  |  |  |  |
| ***Pre-existing conditions*** |  |  |  |  |  |  |  |  |  |  |  |  |  |  |  |  |  |
| None | 104 | 68.4 |  | 22 | 75.9 |  | 9 | 56.3 |  | 22 | 73.3 |  | 25 | 83.3 |  | 26 | 65.0 |
| Bronchopulmonary dysplasia | 6 | 3.9 |  | 1 | 3.4 |  | 2 | 12.5 |  | 2 | 6.7 |  | 0 | 0.0 |  | 1 | 2.5 |
| Laryngeal or tracheobronchial anomaly | 9 | 5.9 |  | 1 | 3.4 |  | 1 | 6.3 |  | 1 | 3.3 |  | 1 | 2.7 |  | 5 | 12.5 |
| Any respiratory tract disease | 33 | 21.7 |  | 4 | 13.8 |  | 6 | 37.5 |  | 6 | 20.0 |  | 8 | 21.6 |  | 9 | 22.5 |
| Congenital heart disease** | 8 | 5.3 |  | 1 | 3.4 |  | 1 | 6.3 |  | 2 | 6.7 |  | 3 | 8.1 |  | 1 | 2.5 |
| Neuromuscular disease | 7 | 4.6 |  | 1 | 3.4 |  | 0 | 0.0 |  | 0 | 0.0 |  | 3 | 8.1 |  | 3 | 7.5 |
| Immunocompromised state or malignancy | 0 | 0 |  | 0 | 0.0 |  | 0 | 0.0 |  | 0 | 0.0 |  | 0 | 0.0 |  | 0 | 0.0 |
| Down syndrome | 4 | 2.6 |  | 0 | 0.0 |  | 1 | 6.3 |  | 0 | 0.0 |  | 3 | 8.1 |  | 0 | 0.0 |
| Other | 12 | 7.9 |  | 1 | 3.4 |  | 1 | 6.3 |  | 2 | 6.7 |  | 3 | 8.1 |  | 5 | 12.5 |
| ≥1 condition | 48 | 32.0 |  | 7 | 24.1 |  | 7 | 43.8 |  | 8 | 26.7 |  | 12 | 32.4 |  | 14 | 35.0 |
|  |  |  |  |  |  |  |  |  |  |  |  |  |  |  |  |  |  |
| ***In-hospital diagnosis and management*** | |  |  |  |  |  |  |  |  |  |  |  |  |  |  |  |  |
|  | |  |  |  |  |  |  |  |  |  |  |  |  |  |  |  |  |
| Main respiratory tract discharge diagnosis | |  |  |  |  |  |  |  |  |  |  |  |  |  |  |  |  |
| Upper respiratory tract infection | 8 | 5.3 |  | 1 | 3.4 |  | 0 | 0.0 |  | 1 | 3.3 |  | 1 | 2.7 |  | 5 | 12.5 |
| Wheezy bronchitis | 6 | 3.9 |  | 0 | 0.0 |  | 1 | 6.3 |  | 2 | 6.7 |  | 2 | 5.4 |  | 1 | 2.5 |
| Bronchiolitis | 116 | 76.3 |  | 24 | 82.8 |  | 12 | 75.0 |  | 25 | 83.3 |  | 33 | 89.2 |  | 22 | 55.0 |
| Pneumonia*** | 22 | 14.5 |  | 4 | 13.8 |  | 3 | 18.8 |  | 2 | 6.7 |  | 1 | 2.7 |  | 12 | 30.0 |
|  |  |  |  |  |  |  |  |  |  |  |  |  |  |  |  |  |  |
| Supplemental O2 administration mode |  |  |  |  |  |  |  |  |  |  |  |  |  |  |  |  |  |
| None | 3 | 2.0 |  | 0 | 0.0 |  | 0 | 0.0 |  | 0 | 0.0 |  | 0 | 0.0 |  | 3 | 7.5 |
| Nasal cannula or funnel | 11 | 7.2 |  | 0 | 0.0 |  | 3 | 18.8 |  | 0 | 0.0 |  | 4 | 10.8 |  | 4 | 10.0 |
| HFNC | 87 | 57.2 |  | 20 | 69.0 |  | 11 | 68.8 |  | 16 | 53.3 |  | 25 | 67.6 |  | 15 | 37.5 |
| CPAP | 28 | 18.4 |  | 1 | 3.4 |  | 1 | 6.3 |  | 9 | 30.0 |  | 5 | 13.5 |  | 12 | 30.0 |
| Mechanical ventilation | 23 | 15.1 |  | 8 | 27.6 |  | 1 | 6.3 |  | 5 | 16.7 |  | 3 | 8.1 |  | 6 | 15.0 |
|  |  |  |  |  |  |  |  |  |  |  |  |  |  |  |  |  |  |
| Antimicrobial therapy for |  |  |  |  |  |  |  |  |  |  |  |  |  |  |  |  |  |
| Acute otitis media | 5 | 3.3 |  | 1 | 3.4 |  | 0 | 0.0 |  | 1 | 3.3 |  | 2 | 5.4 |  | 1 | 2.5 |
| Pneumonia | 42 | 27.6 |  | 13 | 44.8 |  | 5 | 31.3 |  | 5 | 16.7 |  | 6 | 16.2 |  | 13 | 32.5 |
| Sepsis/fever without source | 21 | 13.8 |  | 5 | 17.2 |  | 3 | 18.8 |  | 5 | 16.7 |  | 5 | 13.5 |  | 3 | 7.5 |
| Other/unrelated to RSV | 11 | 7.2 |  | 2 | 6.9 |  | 1 | 6.3 |  | 4 | 13.3 |  | 2 | 5.4 |  | 2 | 5.0 |
| None | 73 | 48.0 |  | 8 | 27.6 |  | 7 | 43.8 |  | 15 | 50.0 |  | 22 | 59.5 |  | 21 | 52.5 |
|  |  |  |  |  |  |  |  |  |  |  |  |  |  |  |  |  |  |
| ***Outcome*** | | | | | | | | | | | | | | | | | |
| Hospital stay (d) [IQR] | 8.0  [6.0-11.0] |  |  | 8.1  [5.7-9.0] |  |  | 8.5  [7.0-13.5] |  |  | 7.8  [6.1-9.9] |  |  | 7.9  [6.0-11.0] |  |  | 10.0  [7.0-16.0] |  |
| Death | 2 | 1.3 |  | 0 |  |  | 0 |  |  | 0 |  |  | 0 |  |  | 2 | 5.0 |
| \| * 10 of 40 (25%) ICU cases in 2023-2024 vs. 11 of 112 (9.8%) ICU cases in 2018-2023 (OR 3.06, 95% CI 1.86-7.90; p=0.034). \| \| --- \| | | | | | | | | | | | | | | | | | |
| ** hemodynamically significant at the time of RSV hospitalization. | | | | | | | | | | | | | | | | | |
| *** 12 of 40 (30.0%) ICU cases in 2023-2024 vs. 10 of 122 (8.9%) ICU cases in 2018-2023 (OR 4.37; 95% CI 1.71-11.16; p=0.003). | | | | | | | | | | | | | | | | | |

**Table S2.** Clinical characteristics of 236 RSV hospitalizations occurring in children aged 12 to 23 months between 2018 an 2024 at the Department of Pediatrics, Bern University Hospital, Bern, Switzerland

|  | **2018-2024** | |  | **2018-2019** | |  | **2019-2020** | |  | **2021-2022** | |  | **2022-2023** | |  | **2023-2024** | |
| --- | --- | --- | --- | --- | --- | --- | --- | --- | --- | --- | --- | --- | --- | --- | --- | --- | --- |
|  | **n** | **%** |  | **n** | **%** |  | **n** | **%** |  | **n** | **%** |  | **n** | **%** |  | **n** | **%** |
| ***Demography*** |  |  |  |  |  |  |  |  |  |  |  |  |  |  |  |  |  |
| All cases | 1’339 |  |  | 236 |  |  | 180 |  |  | 306 |  |  | 299 |  |  | 318 |  |
| Cases 12-23 months of age | 236 | 17.6 |  | 43 | 18.2 |  | 30 | 16.7 |  | 51 | 16.7 |  | 52 | 17.4 |  | 60 | 18.9 |
| Male sex (n) | 130 | 54.9 |  | 25 | 56.8 |  | 18 | 62.1 |  | 29 | 52.7 |  | 27 | 55.1 |  | 31 | 51.7 |
|  |  |  |  |  |  |  |  |  |  |  |  |  |  |  |  |  |  |
| Gestational age (n=176) |  |  |  |  |  |  |  |  |  |  |  |  |  |  |  |  |  |
| < 32 weeks | 24 | 13.4 |  | 8 | 20.0 |  | 4 | 20.0 |  | 2 | 4.8 |  | 2 | 5.0 |  | 8 | 21.6 |
| 32-36 weeks | 21 | 11.7 |  | 8 | 20.0 |  | 1 | 5.0 |  | 4 | 9.5 |  | 2 | 5.0 |  | 6 | 16.2 |
| ≥ 37 weeks | 131 | 73.2 |  | 23 | 57.5 |  | 16 | 80.0 |  | 33 | 78.6 |  | 38 | 95.0 |  | 21 | 56.8 |
|  |  |  |  |  |  |  |  |  |  |  |  |  |  |  |  |  |  |
| Birth weight (gm) (n=155) |  |  |  |  |  |  |  |  |  |  |  |  |  |  |  |  |  |
| < 750 | 9 | 5.6 |  | 5 | 12.8 |  | 1 | 5.6 |  | 1 | 2.6 |  | 1 | 2.8 |  | 1 | 3.4 |
| 750-1499 | 11 | 6.9 |  | 2 | 5.1 |  | 3 | 16.7 |  | 1 | 2.6 |  | 1 | 2.8 |  | 4 | 13.8 |
| 1500-2499 | 19 | 11.9 |  | 6 | 15.4 |  | 1 | 5.6 |  | 3 | 7.7 |  | 3 | 8.3 |  | 6 | 20.7 |
| ≥ 2500 | 116 | 72.5 |  | 25 | 64.1 |  | 14 | 77.8 |  | 30 | 76.9 |  | 31 | 86.1 |  | 16 | 55.2 |
|  | 155 |  |  |  |  |  |  |  |  |  |  |  |  |  |  |  |  |
| ***Pre-existing conditions*** |  |  |  |  |  |  |  |  |  |  |  |  |  |  |  |  |  |
| None | 158 | 66.7 |  | 22 | 50.0 |  | 21 | 72.4 |  | 32 | 58.2 |  | 41 | 83.7 |  | 42 | 70.0 |
| Bronchopulmonary dysplasia | 16 | 6.8 |  | 6 | 13.6 |  | 4 | 13.8 |  | 1 | 1.8 |  | 2 | 4.1 |  | 3 | 5.0 |
| Laryngeal or tracheobronchial anomaly | 8 | 3.4 |  | 1 | 2.3 |  | 1 | 3.4 |  | 0 | 0.0 |  | 2 | 4.1 |  | 4 | 6.7 |
| Any respiratory tract disease | 56 | 23.6 |  | 16 | 36.4 |  | 9 | 31.0 |  | 12 | 21.8 |  | 8 | 16.3 |  | 11 | 18.3 |
| Congenital heart disease* | 6 | 2.5 |  | 2 | 4.5 |  | 0 | 0 |  | 3 | 5.5 |  | 0 | 0.0 |  | 1 | 1.7 |
| Neuromuscular disease | 9 | 3.8 |  | 2 | 4.5 |  | 0 | 0 |  | 1 | 1.8 |  | 5 | 10.2 |  | 1 | 1.7 |
| Immunocompromised state or malignancy | 1 | 0.4 |  | 0 | 0.0 |  | 0 | 0 |  | 1 | 1.8 |  | 0 | 0.0 |  | 0 | 0.0 |
| Down syndrome | 3 | 1.3 |  | 1 | 2.3 |  | 0 | 0 |  | 1 | 1.8 |  | 0 | 0.0 |  | 1 | 1.7 |
| Other | 17 | 7.2 |  | 3 | 6.8 |  | 0 | 0 |  | 5 | 9.1 |  | 5 | 10.2 |  | 4 | 6.7 |
| ≥ 1 condition | 78 | 32.9 |  | 21 | 47.7 |  | 9 | 31.0 |  | 19 | 34.5 |  | 11 | 22.4 |  | 18 | 30.0 |
|  |  |  |  |  |  |  |  |  |  |  |  |  |  |  |  |  |  |
| ***In-hospital diagnosis and management*** | | | | | | | | | | | | | | | | | |
|  |  |  |  |  |  |  |  |  |  |  |  |  |  |  |  |  |  |
| Main discharge diagnosis |  |  |  |  |  |  |  |  |  |  |  |  |  |  |  |  |  |
| Upper respiratory tract infection | 26 | 11.0 |  | 3 | 6.8 |  | 3 | 10.3 |  | 12 | 21.8 |  | 5 | 10.2 |  | 3 | 5.0 |
| Wheezy bronchitis | 43 | 18.1 |  | 6 | 13.6 |  | 4 | 13.8 |  | 13 | 23.6 |  | 8 | 16.3 |  | 12 | 20.0 |
| Bronchiolitis | 113 | 47.7 |  | 25 | 56.8 |  | 18 | 62.1 |  | 16 | 29.1 |  | 36 | 73.5 |  | 18 | 30.0 |
| Pneumonia | 54 | 22.8 |  | 9 | 20.5 |  | 5 | 17.2 |  | 10 | 18.2 |  | 3 | 6.1 |  | 27 | 45.0 |
|  |  |  |  |  |  |  |  |  |  |  |  |  |  |  |  |  |  |
| Supplemental oxygen administration | | | | | | | | | | | | | | | | | |
| None | 19 | 8.0 |  | 3 | 6.8 |  | 1 | 3.4 |  | 5 | 9.1 |  | 5 | 10.2 |  | 5 | 8.3 |
| Nasal cannula or funnel | 179 | 75.5 |  | 35 | 79.5 |  | 26 | 89.7 |  | 40 | 72.7 |  | 41 | 83.7 |  | 37 | 61.7 |
| High flow nasal cannula | 33 | 13.9 |  | 5 | 11.4 |  | 3 | 10.3 |  | 4 | 7.3 |  | 6 | 12.2 |  | 15 | 25.0 |
| CPAP | 3 | 1.3 |  | 0 | 0.0 |  | 0 | 0.0 |  | 1 | 1.8 |  | 0 | 0.0 |  | 2 | 3.3 |
| Mechanical ventilation | 2 | 0.8 |  | 0 | 0.0 |  | 0 | 0.0 |  | 1 | 1.8 |  | 0 | 0.0 |  | 1 | 1.7 |
|  |  |  |  |  |  |  |  |  |  |  |  |  |  |  |  |  |  |
| Antimicrobial therapy for |  |  |  |  |  |  |  |  |  |  |  |  |  |  |  |  |  |
| Acute otitis media | 54 | 22.8 |  | 9 | 20.5 |  | 8 | 27.6 |  | 18 | 32.7 |  | 7 | 14.3 |  | 12 | 20.0 |
| Pneumonia | 17 | 7.2 |  | 4 | 9.1 |  | 5 | 17.2 |  | 1 | 1.8 |  | 2 | 4.1 |  | 5 | 8.3 |
| Sepsis/fever without source | 3 | 1.3 |  | 1 | 2.3 |  | 0 | 0.0 |  | 1 | 1.8 |  | 0 | 0.0 |  | 1 | 1.7 |
| Other/unrelated to RSV | 3 | 1.3 |  | 2 | 4.5 |  | 0 | 0.0 |  | 0 | 0.0 |  | 1 | 2.0 |  | 0 | 0.0 |
| None | 159 | 67.1 |  | 27 | 61.4 |  | 17 | 58.6 |  | 31 | 56.4 |  | 42 | 85.7 |  | 42 | 70.0 |
|  |  |  |  |  |  |  |  |  |  |  |  |  |  |  |  |  |  |
| ***Outcome*** | | | | | | | | | | | | | | | | | |
| hospital stay (d) | 4.0  [3.0-6.3] |  |  | 4.8  [3.1-7-1] |  |  | 4.0  [3.0-6.0] |  |  | 3.8  [2.9-5.6] |  |  | 4.0  [3.0-5.0] |  |  | 5.0  [3.0-7.0] |  |
| ICU admission (n) | 21 | 8.9 |  | 2 | 4.5 |  | 3 | 10.3 |  | 3 | 5.5 |  | 3 | 6.1 |  | 10 | 16.7 |
| Death | 1 | 0.4 |  | 0 | 0.0 |  | 0 | 0.0 |  | 0 | 0.0 |  | 0 | 0.0 |  | 1 | 1.7 |
| *hemodynamically significant at the time of RSV hospitalisation | | | | | | | | | | | | | | | | | |

**Table S3.** Factors associated Intensive Care Unit (ICU) admission in patients below 16 years of age hospitalized for acute RSV infection between 2018 an 2024 at the Department of Pediatrics, Bern University Hospital, Bern, Switzerland

| Variable | Admitted to PICU, n (%) | Not admitted to PICU, n (%) | Unadjusted model | | Adjusted model | |
| --- | --- | --- | --- | --- | --- | --- |
|  |  |  | OR (95% CI) | p value | OR (95% CI) | p value |
| Sex |  |  |  | 0.82 |  |  |
| Female | 67 (44%) | 535 (45%) | 1 |  |  |  |
| Male | 85 (56%) | 652 (55%) | 1.04 (0.74-1.47) |  |  |  |
| Age group |  |  |  | < 0.001 |  | < 0.001 |
| Age > 12 months | 38 (25%) | 386 (33%) | 1 |  | 1 |  |
| Age < 3 months | 89 (59%) | 406 (34%) | **2.23 (1.5-3.37)** |  | **8.54 (4.32-18.02)** |  |
| Age 3-5 months | 14 (9%) | 190 (16%) | 0.75 (0.38-1.38) |  | 1.8 (0.75-4.27) |  |
| Age 6-12 months | 11 (7%) | 205 (17%) | 0.55 (0.26-1.05) |  | 1.19 (0.47-2.92) |  |
| Gestational age |  |  |  | 0.001 |  | < 0.001 |
| ≥ 37 weeks | 95 (62%) | 887 (75%) | 1 |  | 1 |  |
| < 32 weeks | 10 (7%) | 43 (4%) | **2.17 (1-4.3)** |  | **4.69 (1.78-11.71)** |  |
| 32-36 weeks | 26 (17%) | 104 (9%) | **2.33 (1.42-3.72)** |  | **2.07 (1.18-3.56)** |  |
| Bronchopulmonary Dysplasia (BPD) |  |  |  | 0.19 |  |  |
| No | 146 (96%) | 1162 (98%) | 1 |  |  |  |
| Yes | 6 (4%) | 25 (2%) | 1.91 (0.7-4.44) |  |  |  |
| Laryngeal and/or tracheobronchial anomaly |  |  |  | 0.06 |  | 0.007 |
| No | 146 (96%) | 1169 (98%) | 1 |  | 1 |  |
| Yes | 6 (4%) | 18 (2%) | 2.67 (0.96-6.48) |  | **4.99 (1.6-14.09)** |  |
| Congenital heart disease |  |  |  | 0.009 |  | 0.007 |
| No | 135 (89%) | 1121 (94%) | 1 |  | 1 |  |
| Not hemodynamically significant | 9 (6%) | 50 (4%) | 1.49 (0.67-2.96) |  | 0.88 (0.34-2.09) |  |
| Hemodynamically significant | 8 (5%) | 16 (1%) | **4.15 (1.66-9.63)** |  | **6.4 (2.03-19.35)** |  |
| Neuromuscular disease |  |  |  | 0.027 |  | 0.1 |
| No | 145 (95%) | 1168 (98%) | 1 |  | 1 |  |
| Yes | 7 (5%) | 19 (2%) | **2.97 (1.14-6.88)** |  | 3.9 (0.87-14.6) |  |
| Down Syndrome |  |  |  | 0.08 |  | 0.8 |
| No | 148 (97%) | 1177 (99%) | 1 |  | 1 |  |
| Yes | 4 (3%) | 10 (1%) | 3.18 (0.86-9.64) |  | 1.37 (0.15-7.93) |  |
| Other chronic disease |  |  |  | 0.13 |  |  |
| No | 140 (92%) | 1130 (95%) | 1 |  |  |  |
| Yes | 12 (8%) | 57 (5%) | 1.7 (0.85-3.14) |  |  |  |
| Main respiratory tract discharge diagnosis |  |  |  | 0.028 |  | 0.5 |
| Bronchiolitis | 116 (76%) | 833 (70%) | 1 |  | 1 |  |
| Wheezy bronchitis | 6 (4%) | 121 (10%) | **0.36 (0.14-0.76)** |  | 0.51 (0.15-1.39) |  |
| Pneumonia | 22 (14%) | 145 (12%) | 1.09 (0.65-1.74) |  | 1.1 (0.52-2.2) |  |
| Upper respiratory tract infection | 8 (5%) | 88 (7%) | 0.65 (0.29-1.3) |  | 0.67 (0.24-1.62) |  |
| Antibiotic therapy during hospitalization |  |  |  | < 0.001 |  | < 0.001 |
| No | 73 (48%) | 949 (80%) | 1 |  | 1 |  |
| Yes | 79 (52%) | 238 (20%) | **4.32 (3.05-6.12)** |  | **5.93 (3.86-9.18)** |  |
| Respiratory virus co-infection |  |  |  | 0.20 |  |  |
| No | 137 (90%) | 1105 (93%) | 1 |  |  |  |
| Yes | 15 (10%) | 82 (7%) | 1.48 (0.8-2.56) |  |  |  |

**Table S4.** Comparison of clinical variables among RSV patients diagnosed with pneumonia in 2018-2023 vs. 2023-2024

| Variable | 2018-2023 | 2023-2024 | statistical analysis |
| --- | --- | --- | --- |
| All patients (n) | 1021 | 318 |  |
|  |  |  |  |
| Patients with pneumonia (n, %) | 91 (8.9) | 76 (23.9) | OR 3.21 (95% CI 2.29-4.49) |
| Male sex (n, %) | 55 (60.4) | 41 (53.9) | n.s. |
| Age (median, IQR) | 1.3 [0.3-2.4] | 2.0 [1.4-3.4] | p=0.0001 |
| Presence of comorbidity (n, %) | 24 (26) | 27 (36) | n.s. |
| Duration of hospital stay (median, IQR) | 5.0 [3.6-7.0] | 5.0 [3.0-8.0] | n.s. |
| Maximum C-reactive protein (CR) mg/L (median, IQR) | 43 [14-95] | 44 [12-143] | n.s. |
| Respiratory viral co-infection (n, %) | 7 (7.7) | 9 (11.8) | n.s. |
| Antibiotic therapy (n, %) | 37 (40.7) | 34 (44.7) | n.s. |
| ICU admission (n, %) | 10 (11.0) | 12 (15.8) | n.s. |
| Mechanical ventilation (n, %) | 4 (4.4) | 3 (3.9) | n.s. |
| Death (n, %) | 0 | 2 (2.6) | n.a. |

**Figure S1.** Age group-specific incidences of RSV hospitalizations per epidemiological year. Panel A displays annual incidences per 1’000 live births recorded between 2018 and 2024 for all RSV hospitalizations in children below 5 years of age. Panel B shows the corresponding incidence for Intensive Care Unit (ICU) admissions.


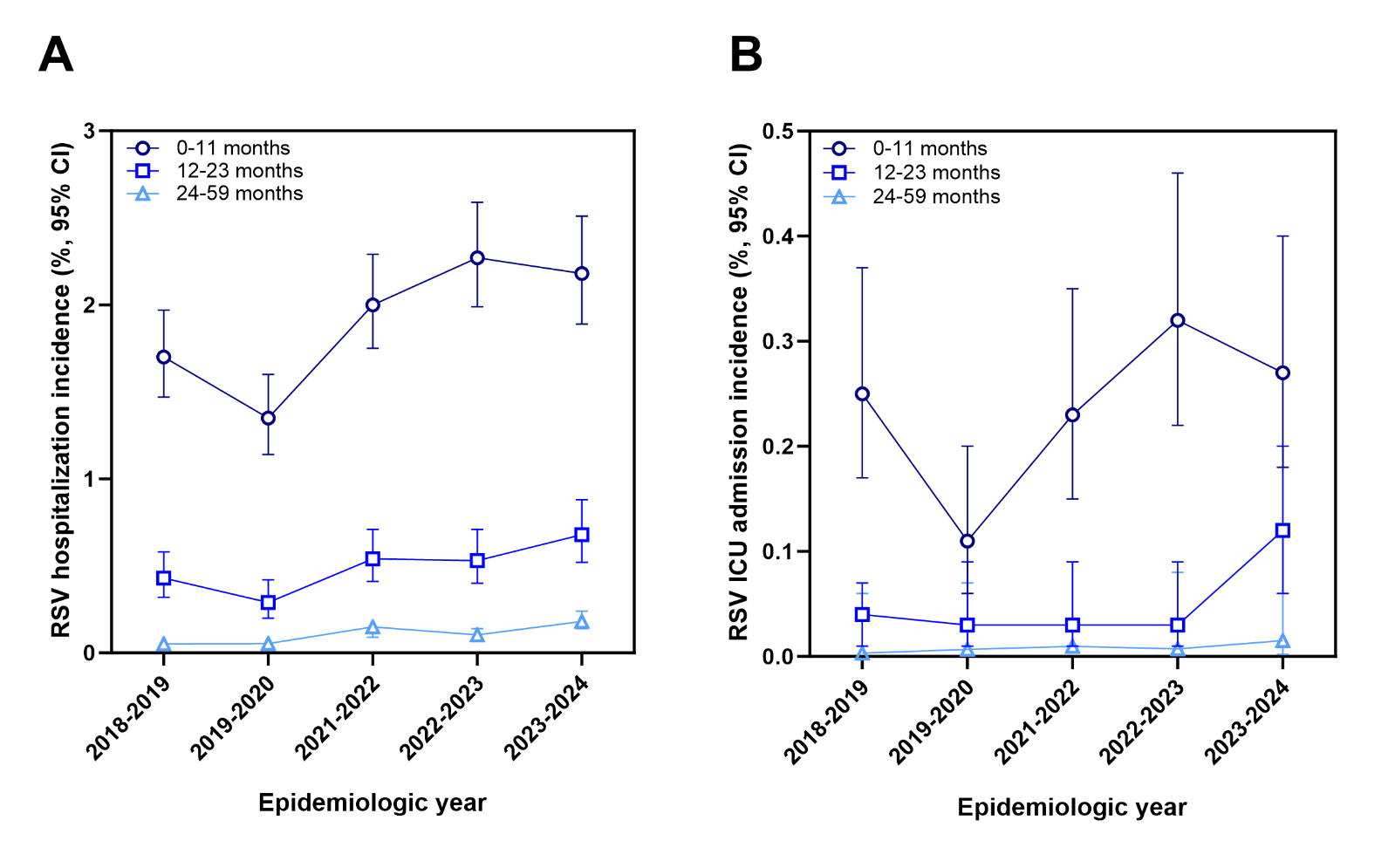

Supplement: Supplementary file 1 — Supplementary file1 (DOCX 350 KB) [file 431_2024_5785_MOESM1_ESM.docx]
